# Eye closure sensitivity as a novel EEG marker of MERRF

**DOI:** 10.1002/epi4.13006

**Published:** 2024-07-06

**Authors:** Douglas R. Nordli, Danielle Natarus, Sho Yano, Douglas R. Nordli

**Affiliations:** ^1^ Comer Children's Hospital University of Chicago Chicago Illinois USA

A 12‐year‐old boy presented with new‐onset convulsions, staring spells, and body twitches. He had mild intellectual disability, necessitating a school individualized education program (IEP). A maternal great aunt had epilepsy, and a maternal grandmother experienced multiple strokes in her early forties. Neurological examination revealed mild imbalance, while physical examination was otherwise unremarkable. Initial electroencephalogram (EEG) findings (Figure 1A) showed a normal background (9 Hz PDR) and evidence of eye closure sensitivity (ECS). The working diagnosis was juvenile myoclonic epilepsy (JME), and the patient started levetiracetam monotherapy. However, despite medication compliance, he continued to experience occasional convulsions and frequent staring spells and myoclonus. A repeat EEG for spell capture was performed, which revealed a slowed posterior dominant rhythm (8 Hz) and a degraded anterior‐to‐posterior gradient compared with the initial EEG, with persistent eye closure sensitivity. No seizures were captured during the recordings. Concerning EEG background changes prompted further investigation into potential mimickers of JME, leading to genetic testing and subsequent mitochondrial testing/trinucleotide repeat testing. Subsequent EEG recording (Figure 1C) demonstrated progressive worsening of background speed and organization. While whole exome sequencing revealed no pathogenic variants, mitochondrial testing identified a pathogenic variant in the *MT‐TK* gene (m.8344A>G), inherited maternally with 96% heteroplasmy, consistent with a diagnosis of a mitochondrial disorder, specifically myoclonic epilepsy with ragged‐red fibers (MERRF).

**FIGURE 1:**
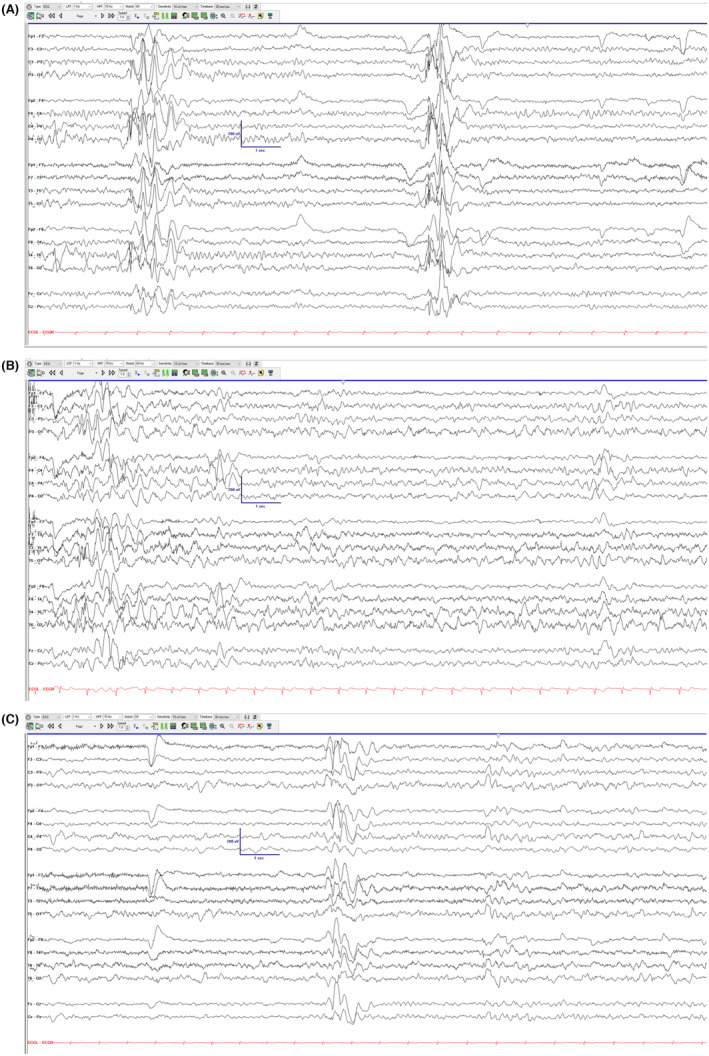
(A) Longitudinal bipolar EEG of initial EEG with ECS and 9 Hz PDR. (B) Longitudinal bipolar electroencephalogram (EEG) of follow‐up EEG 7 months later with slowed PDR (8 Hz) and relative degradation of EEG anterior to posterior gradient. (C) Longitudinal bipolar electroencephalogram (EEG) of follow‐up at 8 months later with further slowing and disorganization of the EEG background.

MERRF is a mitochondrial disease that often affects patients beginning in childhood.^1^ It is caused by pathogenic variants in tRNA genes within the mitochondrial genome, most commonly the *MT‐TK* variant found in our patient.^1^ The disease typically presents with progressive myoclonic epilepsy (PME), cerebellar ataxia, progressive muscle weakness, sensorineural hearing loss, cardiomyopathy or cardiac arrhythmia, and dementia.^2^ The severity of the disease and seizures varies along a phenotypic spectrum, influenced by the percentage of heteroplasmy. Interestingly, the percentage of heteroplasmy in brain tissue is higher than in serum, which correlates with disease severity.^3^ However, brain tissue samples show variable amounts of heteroplasmy, indicating that more research is needed to understand this correlation.^3^ Myoclonus is classically considered photosensitive in many cases. There is a paucity of literature on the detailed electrophysiological features of MERRF. Some authors have noted irregular spike and wave discharges, focal occipital spikes, and abnormal backgrounds.^4^ Currently, there are no FDA‐approved precision therapies for MERRF, so the mainstay of treatment remains symptomatic management. Counseling for patients with MERRF emphasizes the importance of avoiding mitochondrial‐toxic agents, such as valproic acid.^5^ Valproic acid interferes with beta‐oxidation in mitochondria due to its structural similarity to simple fatty acids. This mechanism, among others, is why valproic acid should be avoided in cases of suspected mitochondrial cytopathies.^5^ Patients with MERRF are monitored for symptoms and are particularly at risk for seizures and cardiac arrhythmias.^5^

Progressive myoclonic epilepsy syndromes with ECS have not been previously described. While one report describes a presentation of mitochondrial encephalomyopathy with lactic acidosis and stroke‐like episodes (MELAS) with fixation off sensitivity (FOS), the overall literature on EEG findings in various types of mitochondrial disease is scant.^6^ ECS can be seen in various forms of idiopathic generalized epilepsy syndromes, some self‐limited epilepsies, and cases of structural occipital lobe epilepsy.^7^ Sunflower syndrome has been noted to be associated with ECS in particular.^8^ ECS most often comes to mind in association with epilepsy with myoclonic absences (EMA), where valproic acid is one of the treatments with the highest effectiveness.^7^ Valproic acid is also effective in other syndromes associated with ECS.

Our case highlights a novel and critical finding that PMEs must also be on the differential diagnosis in patients with ECS. While PMEs are rare, recognition of their unique clinical and electrographic features can help to aid detection. Another important point relates to treatment selection. Valproate is an excellent choice for treatment of many epilepsies associated with ECS but could be harmful for patients with mitochondrial cytopathies.^5^ Therefore, if a patient with suspected PME or atypical JME has ECS, a mitochondrial disorder should be considered before initiating treatment with valproate.

While the patient's seizure types and initial EEG findings were initially suggestive of juvenile myoclonic epilepsy, experts note that cognitive impairment stands as an exclusionary criterion. Furthermore, the presence of neurological disorders among maternal family members heightens suspicion for a mitochondrial disorder. Additionally, an abnormal EEG background warrants consideration that the patient may not conform to the idiopathic generalized epilepsy profile but rather may manifest an epileptogenic encephalopathy.^9^ Such patients, as illustrated in this case, often harbor a genetic underpinning and therefore would benefit from genetic testing.^9^ Fortunately, recognition of the clinical and electrographic abnormalities described in this case facilitated a diagnosis within 9 months of epilepsy onset, without exposure to valproic acid therapy. Since timely diagnosis is important in improving the outcome of patients with epileptogenic encephalopathies, close attention to clinical and electrographic details remains pertinent, even in the modern era with widespread availability of genetic testing. Expanding our knowledge of who to test and what tests to order can be beneficial.

## CONFLICT OF INTEREST STATEMENT

None of the authors has any conflict of interest to disclose. We confirm that we have read the Journal's position on issues involved in ethical publication and affirm that this report is consistent with those guidelines.

## ETHICAL APPROVAL

This manuscript preparation and data collection were conducted in accordance with the Declaration of Helsinki. The collection and evaluation of all protected patient health information was performed in a Health Insurance Portability and Accessibilty Act compliant manner. All necessary permissions have been obtained with written permission granted by the copyright owner.

## PATIENT CONSENT STATEMENT

Patient consent was obtained in preparation of this manuscript.

## IRB STATEMENT

This manuscript did not require IRB approval.

## Data Availability

The data that support the findings of this study are available from the corresponding author, DN upon reasonable request.
